# Experimental investigation of process parameters in Wire-EDM of Ti-6Al-4 V

**DOI:** 10.1038/s41598-025-90486-2

**Published:** 2025-02-15

**Authors:** Manoj Jagdale, Nitin Ambhore, Rakesh Chaudhari, Atul Kulkarni, Masuk Abdullah

**Affiliations:** 1 Department of Mechanical Engineering, Vishwakarma Institute of Information Technology, Kondhwa, 411048 Pune India; 2 Department of Mechanical Engineering, School of Technology, Pandit Deendayal Energy University, Raisan, 382007 Gandhinagar India; 3 Department of Mechanical Engineering, Vishwakarma Institute of Technology, Pune, 411037 India; 4 Department of Vehicles Engineering, Faculty of Engineering, University of Debrecen, Ótemető strt. 2-4, Debrecen, 4028 Hungary

**Keywords:** Wire Electrical Discharge Machining, Ti-6Al-4V, Material removal rate, Taguchi Method, Surface roughness, Engineering, Materials science

## Abstract

Wire electric discharge machining (WEDM) is a recent technique that is useful in machining Ti-6Al-4 V alloy, which is a material that is preferred in many industries due to its exceptional hardness. This paper aims to evaluate the effects of WEDM process parameters on the machining characteristics of Ti-6Al-4 V alloy. The 4-axis CNC WEDM machine that was used in this study had brass wire as the electrode and de-ionized water as the dielectric fluid. The parameters under investigation were the peak current (Ip), pulse on time (TON), pulse off time (TOFF), and servo voltage (SV) set at 3 levels each. The experimentation was based on Taguchi’s L9 orthogonal array design. The material removal rate (MRR) and surface roughness of machined ash components were Ra. A total of three Ra results were analyzed using ANOVA. It was shown that response surface methodology, pulse time ton and peak electric current had more significant effects on MRR. Effect-wise results indicated that peak current and time on P ring test allow surface finish to be within MRR levels. It is peak electric current that determines a 72.75% effect on MRR whereas extreme time has an 11.68 balanced effect on peak current. In the case of Ra, peak electric current and extreme pulse time remain dominant factors. The results suggest that higher Ra is favored by less increase in input energy as both peak current and time have been decreased.

## Introduction

Electrical Discharge Machining or EDM is a novel technique for machining that uses rapid and repeated electric sparks that occur between a tool and a workpiece that are both submerged in dielectric fluid to cut materials that conduct electric currents. The process begins with the application of voltage to both the tool and the workpiece. This causes dielectric breakdown and creates a plasma channel. The temporary channel exhibits enormous temperatures that exceed 10,000 K. This results in melting and the evaporation of the materials on both electrodes. Once the discharge stops, the channel collapses while the dielectric fluid removes the eroded materials. Repeating this achieves the desired material removal. For the process of EDM to be optimal, the sparking and gap phenomena are essential. The process incorporates complex interactions such as evaporation, melting, heat conduction, energy distribution, gas bubble formation, and even the collapse of the bubbles in the discharge channel. These interactions are vital to the process and the overall integrity of the machined components. In conclusion, the physics of EDM revolves around the thermal and electrical interactions that result in the needed material removal via melting and vaporization^1^. The most common titanium alloy is titanium 6 − 4, which contains 90% of titanium (Ti), 6% of aluminum & 4% of vanadium^2^. This metal alloy possesses a variety of features, which include a low density combined with high strength, excellent corrosion resistance, and high biocompatibility, allowing it to be widely applied in industries such as aerospace, marine, automotive, medicine, and sports equipment^3^. The alloy Ti-6Al-4 V has a very good strength-to-weight ratio, being an excellent replacement for steel in certain applications where weight is important and offers the same strength whilst weighing almost half the amount^4^. Like most titanium alloys, Ti-6-4is resistant to corrosion and pitting, and this alloy is frequently used in saltwater and acidic conditions, making it suitable for marine and industrial equipment applications. In addition to this, it is biocompatible and resistant to body fluids, making it an excellent candidate for applications such as orthopedic implants and dental fixtures due to its strength and long-lasting nature. The alloy Ti-6Al-4 V has exceptional corrosion resistance in harsh environments such as seawater or acidic solutions which make it a crucial alternative for marine engineering structures and industrial equipment working in corrosive conditions. Its biocompatibility and body fluids tolerance support a wide range of applications of Ti-6Al-4 V, however, in orthopedic and dental implant use it offers a long waiting life and compatibility with the human body. All of these features advance Ti-6Al-4 V material so that they focus on advancing prosthetics, joint replacement, or other replacements of critical medical devices^5^.

**Fig. 1:**
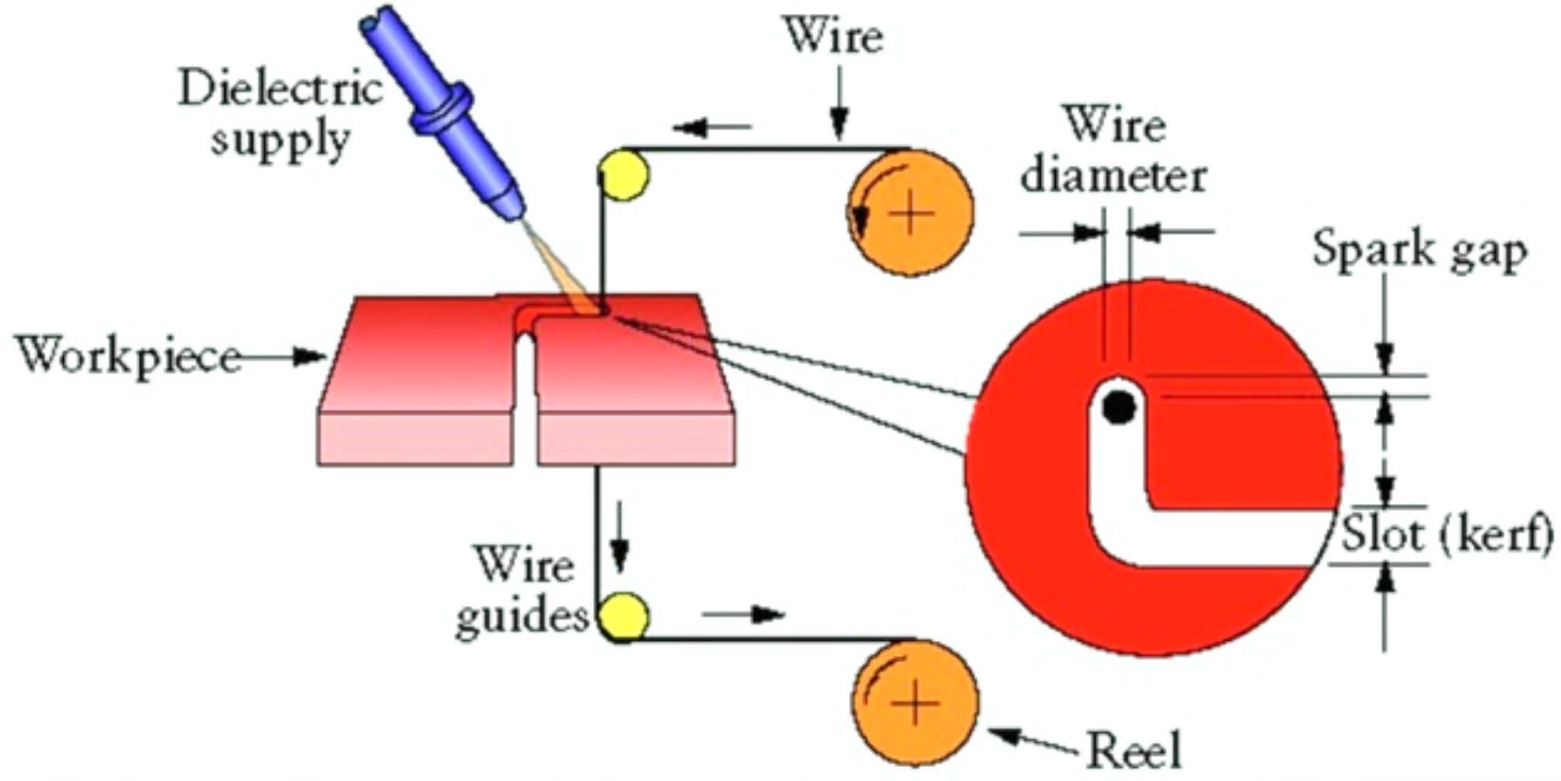
A schematic representation of Wire-EDM process^32^.

Despite its numerous advantages, challenges remain in optimizing the processing and fabrication of Ti-6Al-4 V to ensure cost-effectiveness and maximize performance^6^. The characteristics managed factor separately influence the mechanical parameters of alloy which suggests both precise control and understanding of these factors^7^. Also, well high reactivity of titanium alloy parameters during work with oxygen and nitrogen needs management situations to avoid pollution and the alloy keeps the expected mechanics parameters. As controlled atmosphere needs to be maintained during the processes of casting, forging and welding so that embrittlement and the presence of wire Electrical Discharge Machining (wire EDM) is a high-quality manufacturing process that is effective in the style of cutting and shaping metal and other conductive materials using specific controlled erosive electrical discharges. This is also a non-contact process, however, a wire (usually brass or any other metal) is employed as a thin electrode that continuously rotates around the workpiece to erode it in a uniform and controlled fashion^9^. Eddy with kurts wire EDM is fast and simple but also complex to put into emerging without expensive setup fixtures and hardware for every part making it difficult to efficiently mass produce parts using wire EDM. This may be time-consuming but intense time and labor costs should not be constraining when dealing with large production volumes^10^. Figure 1 shows the schematic reprentation of Wire-EDM process.

Wire EDM is the creation of electrical discharges between a wire electrode.

and the workpiece, wearing away material to make exact cuts. Directed by a Computer Numerical a CNC system usually ensures that the wire follows a programmed path, thus enabling very fine cutting with great precision. Keeping a uniform gap between the wire and workpiece is essential to obtain Precise results, while the process occurs in a dielectric fluid bath, typically deionized water which serves to control discharges and flush away debris. This controlled erosion process produces a high-precision and fine-surface finish with intricate shapes; hence, it is a versatile method extensively used in industries that require complex geometries and high-quality machining^11^.Wire EDM’s cutting parameters, which include pulse-on-time, pulse-off-time, current, wire tension, voltage, dielectric pressure as well as Wire feed rate, play a very critical role in the process’s efficiency and quality. The process has been utilized in the machining of Ti-6Al-4 V alloy because it suits this high-performance material^12^.

Wire EDM can overcome these problems by cutting the alloy accurately enough without including severe mechanical stress or heat-affected zones that degrade material properties. This process not only enhances the machining efficiency and quality of the Ti-6Al-4 V but also minimizes the chances of material damage, like residual stresses or thermal distortion. Apart from this, the compatibility of wire EDM with advanced design software and CNC systems allows automatic and high repeatability, which further contributes towards the production of consistent and reliable parts. Optimizing WEDM parameters for Ti-6Al-4 V ensures very high productivity, precision and surface quality, and consequently reduces costs and preserves all the inherent properties of the material, making it crucial in high-performance applications^13^. Singh et al.^14^ performed an input parameter optimization for the wire-EDM cutting of a 2.5 mm thickness sheet of a Ti6Al4V alloy. An L8 orthogonal array was used to statistically design the tests. The set of input parameters includes wire tension, wire feed, duty cycle, frequency, voltage, current, and capacitance. Studies have been done on cutting speed, surface roughness, taper angle, and kerf breadth. Good outcomes with the optimized process parameters in the confirmation test. The findings showed that there is an acceptable level of inaccuracy within the experimental and anticipated outcomes. Perumal et al.^15^ studied the Ti-6Al-2Sn-4Zr-2Mo alloy’s wire EDM machining performance using the Taguchi-Grey Relational Approach. Using parameters of 10 µs pulse frequency duration, 7 m/min of wire feed, and 12 g of wire tension, the results show that a 0.289 mm3/min material removal rate was found. Likewise, a 2.119 μm surface roughness was obtained with 6 µs pulse length, 3 m/min wire feed, and 8 g wire tension. Ahmed et al.^16^ discovered that the surface quality and metal removal rate for this alloy were improved by adding Ag, Si, or Ag + Si particles to the dielectric fluid. When Ag powder was added to the dielectric fluid instead of Si or Ag + Si powders, the Material Removal Rate (MRR) increased, and the SR decreased. The most useful parameters on MRR and SR are pulse current and concentration, followed by powder type, pulse on, and pulse off. Ibrahim et al.^17^ examined the impact of TiN-coated Ti6Al4V alloy electro-erosion (EDM) processing parameters using physical vapor deposition (PVD). Using a current of 12 A, tone 100, and Cu electrodes, analysis of variance (ANOVA) was used to identify the most suitable parameters of the MRR value.

Ram Prasad et al.^18^ examined how process factors affected the lead-induced Ti-6Al-4 V alloy’s performance properties. ANOVA has been successfully used to highlight the impact of the process parameters linked to each performance feature, specifically the minimization of SR and DD and the maximization of MRR. Sharma et al.^19^ studied how productivity, as determined by material removal rate (MRR) and surface integrity, was affected by controlled machining factors such as spark on-time (Ton), spark off-time (Toff), servo voltage (Sv), and peak current (Ip). For Ti-6Al-7Nb, an alloy of titanium free of the catalytic ingredient vanadium, the study aims to do a wire-electric discharge processing feasibility analysis. Maddu et al.^20^ studied three elementary techniques—the Multi-Angle Optimisation Technique (MAOT), Information Divergence, and -nD angle - are presented to compare the outcomes with the Multi-Objective Optimisation of the Ratio Analysis (MOORA). By milling titanium alloy (Ti-6Al-4 V) with three distinct electrodes, the EDM output characteristics were determined. Czelusniak et al.^21^ examined the input parameters such as current (Amps), pulse-on (µs), pulse-off (µs), and flushing pressure (Bar) for various electrode materials—brass, bronze, and copper which might be optimized to identify the ideal electrode for machining the Ti-6Al-4 V workpiece. Omarov et al.^22^ discovered that when 10 nF and 90 V are used the maximum MRR of 1.72 × 10^−2^ mm^3^/s was observed, while at 1 nF and 90 V, the smallest SR of 0.309 μm was attained. Additionally, the impact of gap voltage on MRR and SR was significantly less than that of capacitance. Galati et al.^23^ found Wire-EDM is particularly beneficial for machining Ti6Al4V, a titanium alloy that has high strength, low thermal conductivity, and reactivity, which are all difficult to machine in conventional machining. Wire-EDM does not involve direct contact between the tool and workpiece, thus eliminating mechanical stresses and tool wear, and allowing for the precise machining of complex geometries with tight tolerances. It is effective for difficult-to-machine materials, such as Ti6Al4V, achieving high precision and surface finish without inducing mechanical stresses.

Optimal Wire Electric Discharge Machining (WEDM) process parameters such as wire tension, pulse duration, and voltage, in their studies to machine Ti-6Al-4 V alloy, were also obtained by Singh et al. and Farooq et al.^24,25^. Their studies showed that these parameters if optimized greatly increase MRR and surface finish. Singh et al. stated that wire tension plays a significant role in stabilizing the process by wire breakages and improving the shape accuracy. Likewise, Farooq et al. worked on pulse duration and voltage to input energy optimally to ensure high MRR and still maintain the surface quality. These results accentuate the need for such tuning of the parameters in WEDM to overcome the difficulties in machining Ti-6Al-4 V and to make high-quality components for important applications. Altin et al.^26^ optimized WEDM parameters for Ti-6Al-4 V alloy, finding that pulse on/off and peak current were the most significant factors affecting machining efficiency, as measured by Volume Material Removal Rate (VMRR) and Ra. They developed regression equations to establish correlations between the process variables and output characteristics, contributing to a better understanding of the machining process. Collectively, these studies underscore the importance of parameter optimization in achieving efficient WEDM processes across various alloys, providing valuable insights for researchers and practitioners in the field. Vora et al.^27^ investigates the application of Wire Electric Discharge Machining (WEDM) in the machining of titanium alloy, specifically Ti6Al4V, which has remarkable mechanical properties including high strength, and corrosion resistance, and is widely used in industries, but is difficult to machine using conventional processes, is examined in this study. The study was limited to three input factors specified as current, pulse-on duration (Ton), and pulse-off duration (Toff), while material removal rate (MRR) and surface roughness (SR) were considered to be the two most important output measures. Empirical models were built to correlate the inputs and the output responses through Taguchi’s L9 orthogonal array for experimental design. Good confirmation of the models was provided using ANOVA, revealing useful information on how parameter combinations can be adjusted to achieve satisfactory machining production rates and surface finish. Muthuramalingam et al.^28^ analyze the optimization of WEDM parameters during the machining of Inconel 718, which is a superalloy with high strength and heat resistance. In their research work, the authors applied the Taguchi-based Grey Relational Analysis (TGRA) method and focused on performance characteristics such as material removal rate, surface roughness, and dimensional accuracy. The authors were able to demonstrate the efficiency of the TGRA method in determining grade machining parameters which leads to higher productivity and accuracy during manufacturing operations. This has significant importance for industries that rely on machining difficult materials with high performance. Muthuramalingam et al.^29^ studied the impact of energy distribution and process parameters on the tool wear during the Electrical Discharge Machining (EDM) process. It was noted in the study that such energy highly contributes to inefficient EDM operations, thus it is vital to control the energy given into the system while optimizing parameters like pulse current, pulse duration, and voltage. The findings reveal how the energy distribution during the EDM process can be further improved relative to the degree of tool erosion. This study is critical for precision-cut EDM operations at a low-cost budget. Muthuramalingam et al.^30^applied the Assignments of Weight Method to study the multi-response optimization of EDM process parameters. The critical parameters like pulse current, pulse duration, and voltage were optimized to strike a balance of performance metrics like material removal rate, surface roughness, and tool wear rate. The authors have demonstrated the application of the weighting method in giving priority to the performance characteristics and hence offered a systematic approach for the improvement of EDM efficiency and quality of output.

Nonetheless, research into improving these aspects is ongoing, to reduce production costs and expand the range of applications for this exceptional alloy. It can be noticed from the literature that advanced Wire EDM (WEDM) parameters for Ti-6Al-4 V alloy have been optimized to a higher level yet some core aspects of the investigation remain unexplored. There is plenty of evidence to suggest the niche studies in existence mostly skew toward single-objective optimizations. There is also evidence to suggest that such studies do not advocate for multi objective approaches that consider MRR, surface finish and tool wear. In addition, other studies employ sophisticated modeling tools like machine learning that are not so prevalent while also the most obvious essential process parameters including wire tensions and dielectric fluid characteristics are frequently overlooked. Integration of the sustainability angles into optimization works such as energy efficiency or even the carbon footprint is very seldom made. On top of that, the majority of the parameter variance addressed in the experimental investigations is narrow, therefore limiting the applicability of the findings under real industrial conditions; however, specific challenges of Ti-6Al-4 V including wire breakage and thermal damage are still insufficiently covered.

The scope of the present studies has been framed in as follows.

- To examine the effect of WEDM (Wire electric discharge machining) process wires current, pulse on time, pulse off time and servo voltage on the machining performance of Ti-6Al-4 V alloy.
- To determine the impact of WEDM parameters on material removal rate and surface roughness using artificial methods.
- To determine the factors that have significant statistical effects on MRR and Ra using (ANOVA) analysis of variance.
- To estimate WEDM process parameters based on Taguchi’s L9 orthogonal array to have both high values of MRR and moderate values of surface roughness.
- To elucidate the fundamentals of Ti-6Al-4 V alloy machining for its utilization in aerospace and biomedical industry applications.

The research focuses on experimental investigation of the process parameters in WEDM of Ti-6Al-4 V with particular focus on optimizing both material removal rate (MRR) and surface quality.

## Materials and methodology

The experimental setup for investigating the machining process involved a comprehensive series of cuts utilizing a sophisticated four-axis CNC WEDM machine, specifically the Maxicut-E model from manufacturer Electronica Machine Tools Limited India. This WEDM setup consists of crucial components such as the power supply unit, the machine tool, and the dielectric unit, each contributing to the precision and efficiency of the machining process. The workpiece is made of Ti-6Al-4 V alloy with dimensions of 100 × 100 × 12 mm, and was securely fixed onto the worktable using clamps and bolts to ensure stability during machining operations. The chemical composition of Ti-6Al-4 V alloy is: 89% titanium, 6.11% aluminium, 4.2% vanadium and 0.25% iron. The positive and negative terminals of the power supply were meticulously connected to the workpiece and the wire electrode, respectively, enabling the controlled flow of electrical current necessary for the erosion process to take place. It’s worth noting that the machine was equipped with an ISO-frequent pulse generator capable of delivering a maximum current of 30 A, which allowed for precise control over the machining parameters, ensuring consistent and reliable results. During the experiment, a total cutting path length of 819 mm was conducted, providing a substantial amount of data for evaluating the performance and capabilities of the WEDM system under various conditions and settings.

In the EDM (Electrical Discharge Machining) process, performance measures such as Material Removal Rate (MRR) and Ra (Surface Roughness) are directly influenced by variations in process parameters like discharge energy, pulse duration, and gap voltage. As these parameters are adjusted, the interaction between the tool electrode and the workpiece alters the discharge characteristics, thereby impacting the heat generated at the spark gap. A higher MRR is typically associated with higher discharge energy, as there is more material removal per unit time, but this can also result in a rougher surface (higher Ra) due to the larger, more irregular discharge craters. In contrast, reducing the discharge energy tends to improve surface finish (lower Ra) but can decrease MRR. The EDM process is highly sensitive to these variations because the characteristics of the discharge pulse determine the material removal mechanism as well as surface integrity.

In consequence, MRR and SR balance become vital to achieve efficient machining and the required quality. Doreswamy et al.^31^ investigated the Material removal in WEDM of Ti-6Al-4 V alloy is performed through several rapid electrical discharges that occur between the wire electrode and the workpiece. The intense heat generated by each discharge melts and vaporizes a small volume of material from both the electrode and the workpiece, forming a plasma channel. The molten material is expelled out of the machining zone by the dielectric fluid, which also cools and solidifies the remaining material, leaving behind a small crater. The cumulative effect of these discharges results in the progressive erosion of the workpiece surface. The efficiency of this process is influenced by parameters such as peak current, pulse duration, and dielectric fluid properties.

For example, increasing the peak current will enhance the MRR but will likely result in a rougher surface finish due to larger discharge craters. In contrast, the optimization of pulse duration will enhance the quality of the surface due to increased cooling and solidification time between the discharges. Recent studies have explored the influence of these parameters on MRR and surface characteristics, thus providing insight into the complex interplay between process settings and machining outcomes.

**Fig. 2:**
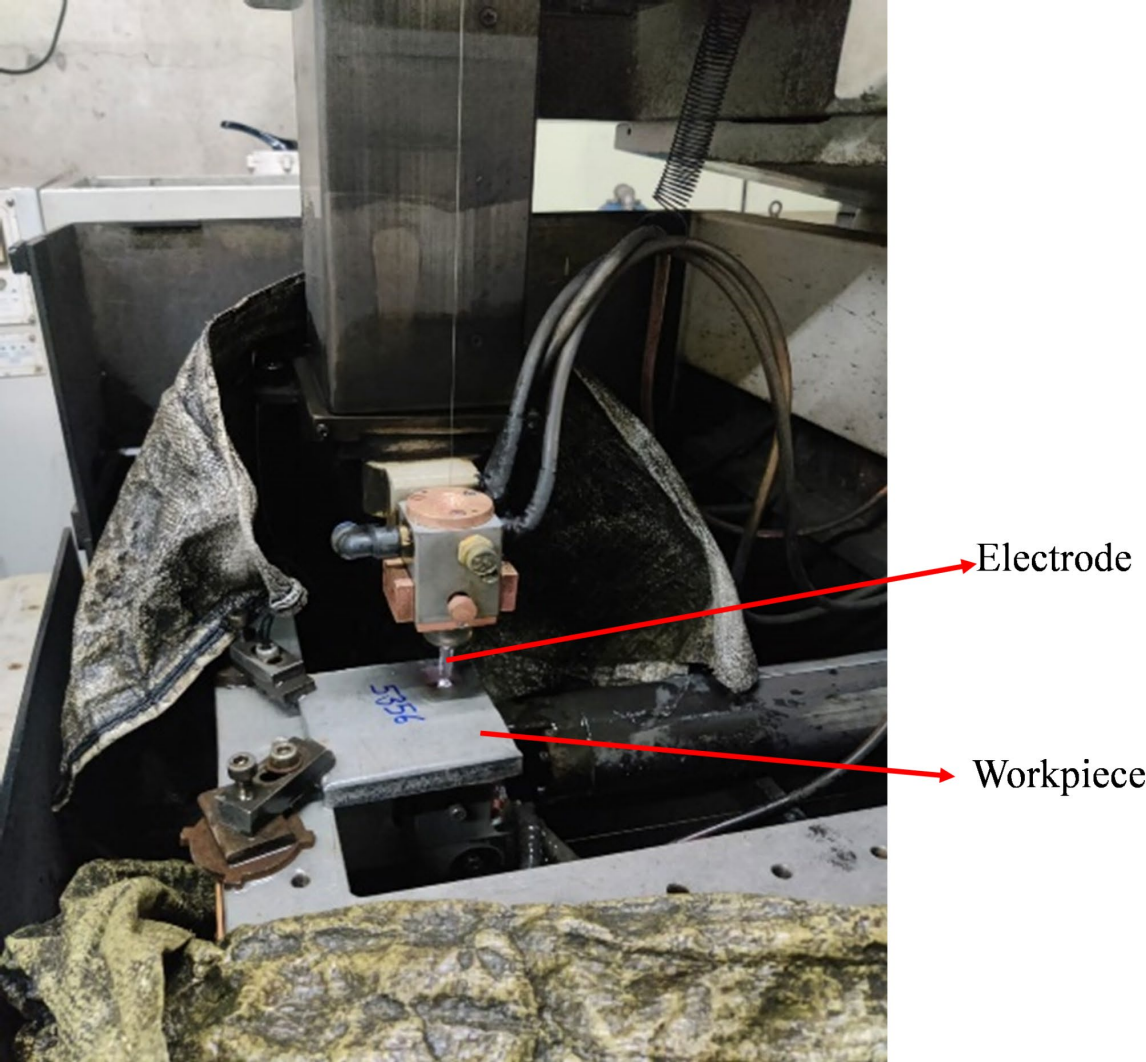
Experimental Setup.

Figure 2 depicted the mounting of the workpiece on a workbench. The EDM Sprintcut machine operates with an accuracy of within ± 10 microns and features a bed size of 300 × 400 mm with a bed height of 220 mm, making it well-suited for precision applications and experimental research.

The four process variables that are considered in this study are Peak current (Ip), Pulse on time (T_ON_), Pulse off time (T_OFF_), and Servo voltage (SV), each maintained at three different levels. The selected levels are shown in Table 1.

**Table 1:** Machining parameters and their respective control factors level.

Control Factors	Level 1	Level 2	Level 3	Units
Peak Current (I_p_)	20	25	30	A
Pulse on Time (T_ON_)	110	115	120	µs
Pulse on Time (T_OFF_)	50	55	60	µs
Servo Voltage (SV)	220	230	240	V

Nine distinct parameter combinations are obtained from the three stages of testing that each factor underwent. To determine how each parameter affected MRR and Ra, these combinations were methodically investigated. The testing strategy using the L9 orthogonal array design is tabulated in Table 2.

**Table 2:** Taguchi L9 Orthogonal Method.

Run	I_*p*_	T_ON_	T_OFF_	SV
1	20	110	50	220
2	20	115	55	230
3	20	120	60	240
4	25	110	55	240
5	25	115	60	220
6	25	120	50	230
7	30	110	60	230
8	30	115	50	240
9	30	120	55	220

**Fig. 3:**
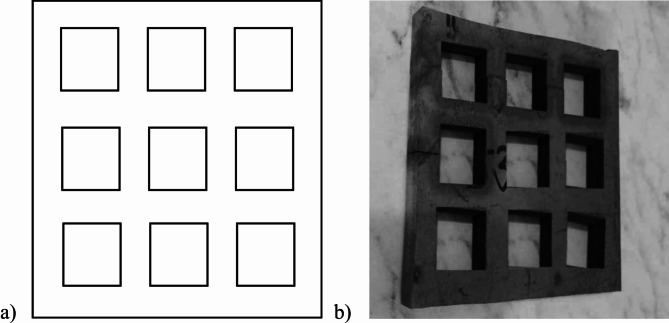
(**a**) Cutting path generated, (**b**) Cuts made on Ti-6Al-4 V.

Firstly, a cutting path was decided in order to make the nine cuts properly on the workpiece, as shown in Fig. 3(a). In order to make 9 equal square cuts on the workpiece, square cuts of size 20 × 20 × 12 mm were selected to be made on the workpiece. Then the path was generated on AutoCad Autodesk software and later it was imported onto the ELCAM software, where the origin was selected so that the CNC machine understands the coordinates from where it should start and also the clearance was kept to be 0.02 mm. Figure 3(b) shows the cuts made on Ti-6Al-4 V.

A standard-sized brass wire electrode, measuring 0.25 mm in diameter, is used for the WEDM process during the experiment. The electrode is immersed in a bath of deionized water, which serves as the dielectric fluid. This liquid helped in cleaning the cutting area of debris and regulating electrical discharges. Other process parameters, such as wire tension, wire feed rate, and flushing pressure, were held constant while four parameters — Peak Current (Ip), Pulse on Time (T_ON_), Pulse off Time (T_OFF_), and Servo Voltage (SV)—were chosen for the experiment.

The MRR is estimated using Eq. (1).

MRR = Cutting Speed (V_c_) × Wire Diameter × Thickness of workpiece (1).

In the experiments, the average surface roughness was measured in terms (Ra). Ra represents the average height of the surface’s profile above and below the mean line. R_a_ was measured using a surface Roughness Tester. The average R_a_ of all four sides of a square cut was taken to determine the surface roughness of every cut.

## Results and discussion

Table 3 displays the outcomes of the experiments. There were nine trials carried out using the Taguchi L9 Method, yielding nine distinct MRR and Ra results. Analysis of Variance (ANOVA) was implemented to assess the impacts of different Wire EDM process cutting parameters on MRR and Ra to analyze and study the experiment outcomes.

**Table 3:** Results of the experiments conducted by the Taguchi L9 method.

S. No	Peak Current	Pulse on time	Pulse off time	Servo Voltage	MRR	*R*_a_(µm)
1	20	110	50	220	7.87	2.14
2	20	115	55	230	7.98	2.19
3	20	120	60	240	8.08	2.23
4	25	110	55	240	8.22	2.47
5	25	115	60	220	8.35	2.66
6	25	120	50	230	8.56	2.84
7	30	110	60	230	8.47	2.56
8	30	115	50	240	8.78	3.01
9	30	120	55	220	8.29	2.79

### Material removal rate

Based on an orthogonal array design, the experimental results shown in Table 4 illustrate the relationship between the relevant signal-to-noise (S/N) ratio and the material removal rate. The ANOVA findings, which examine each parameter’s contribution, are displayed in Table 5. Based on the data presented in the table, it is evident that peak current contributes around 72.75% to the material removal rate, whereas pulse on time contributes around 11.68%.

**Fig. 4:**
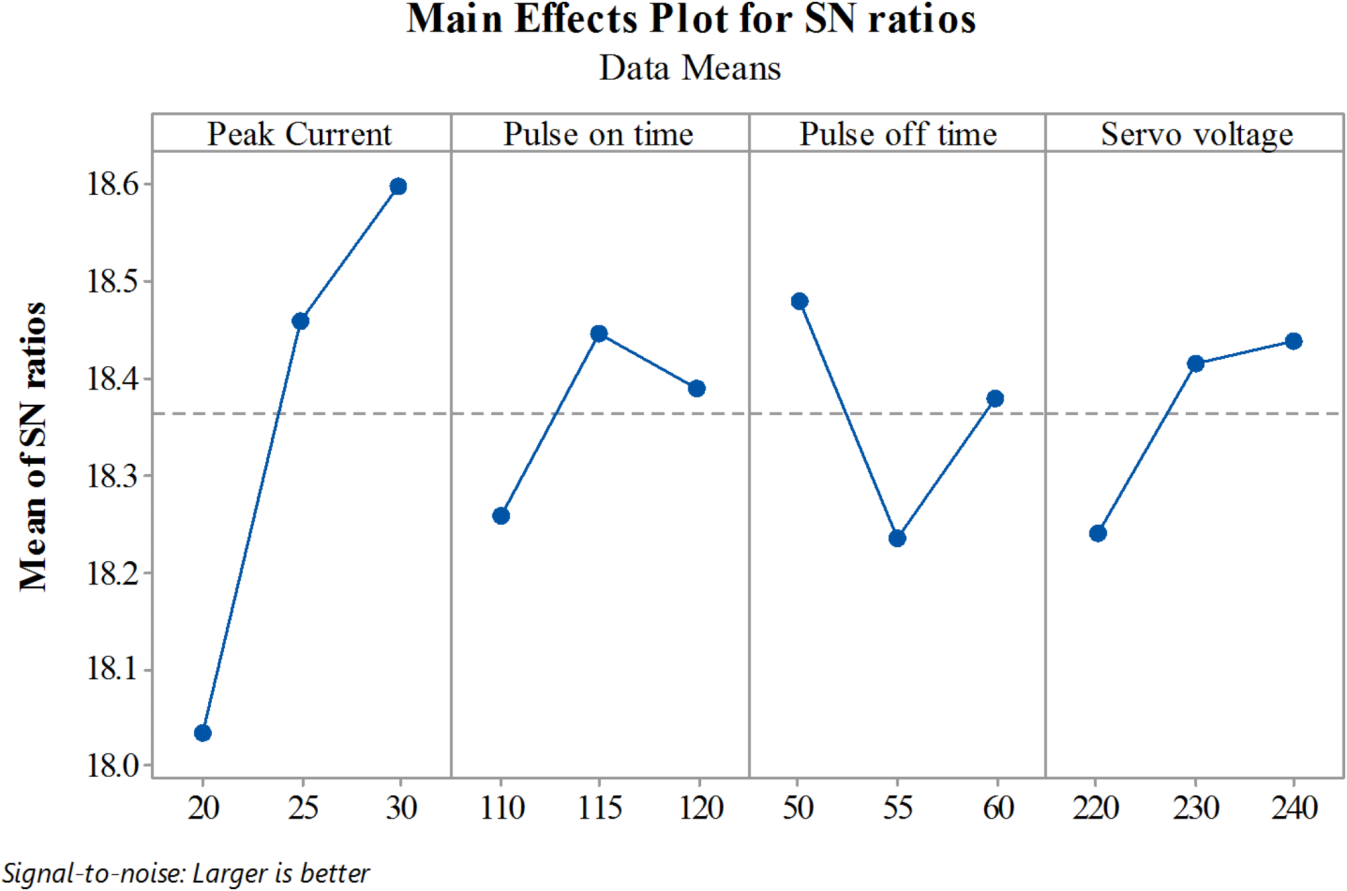
S/N Ratio graphs for Material Removal Rate.

The Material Removal Rate (MRR) rises with increasing the Peak Current (Ip) and Pulse on time (T_ON_), as seen by the S/N response graph in Fig. 4. This is so that the cutting process may continue more quickly since a higher current provides more energy. The cutting rate is further improved by increasing the current, which also increases the energy of the pulse discharge. A higher peak current is essentially necessary to achieve higher material removal rates in Ti-6Al-4 V Wire EDM. Pulse on time (T_ON_) is directly proportional to cutting speed; hence, longer pulse durations lead to faster cutting. On the other hand, parameters like Pulse off time (T_OFF_) and Servo Voltage (SV) contribute less significantly, as their percentage contributions are smaller compared to peak current and pulse on time. The signal-to-noise (S/N) ratio is computed for each combination of factor levels. We have to maximize MRR so we use the larger the better. For a larger-the-better scenario, the S/N ratio is calculated using the following Eq. (2).

S/*N* = − 10 * log(Σ(1/Y^2^)/n) (2).

**Table 4:** Material removal rate and S/N ratio experimental results.

S. No	Peak Current	Pulse on time	Pulse off time	Servo Voltage	MRR	S/*N*
1	20	110	50	220	7.87	17.9194
2	20	115	55	230	7.98	18.0400
3	20	120	60	240	8.08	18.0843
4	25	110	55	240	8.22	18.2974
5	25	115	60	220	8.35	18.4337
6	25	120	50	230	8.56	18.6494
7	30	110	60	230	8.47	18.5576
8	30	115	50	240	8.78	18.8698
9	30	120	55	220	8.29	18.3710

**Table 5:** Experimental results of ANOVA for material removal rate.

Factors	Level 1	Level 2	Level 3	DOF	Sum of squares	Variance	Contribution
I_P_	18.0179	18.4601	18.5994	2	0.1843	0.0926	72.75
T_ON_	18.2581	18.4448	18.3715	2	0.0176	0.0092	11.68
T_OFF_	18.4795	8.2361	18.3618	2	0.0296	0.0144	8.21
SV	18.2413	18.4156	18.4205	2	0.0208	0.0103	6.94
Total				8	0.2533		100

The results obtained in the following research on optimizing WEDM process parameters for Ti-6Al-4 V alloy are in line with and continue to support previous research done by other authors. Similar studies have pointed out that machining Ti-6Al-4 V is difficult because of its low thermal conductivity and high strength and may cause a poor surface finish as well as significant amounts of tool wear. However, with this study in comparison to other studies, the aspect of elaborating on parameter interactions and the subsequent implication on performance measures such as surface roughness, MRR, and dimensional accuracy is different. For instance, MRR is recognized as a significant performance measurement in machining Ti-6Al-4 V since the alloy has poor thermal conductivity and high strength. In general, analogous to the outcome of this study, Sivaprakasam et al. (2022) have been able to show that MRR increases with pulse-on time but is synonymous with poorer surface quality. Here, in this work, this conclusion is expanded even further by presenting the optimum range that promises an effective balance between MRR and surface quality.

### Surface roughness

**Fig. 5:**
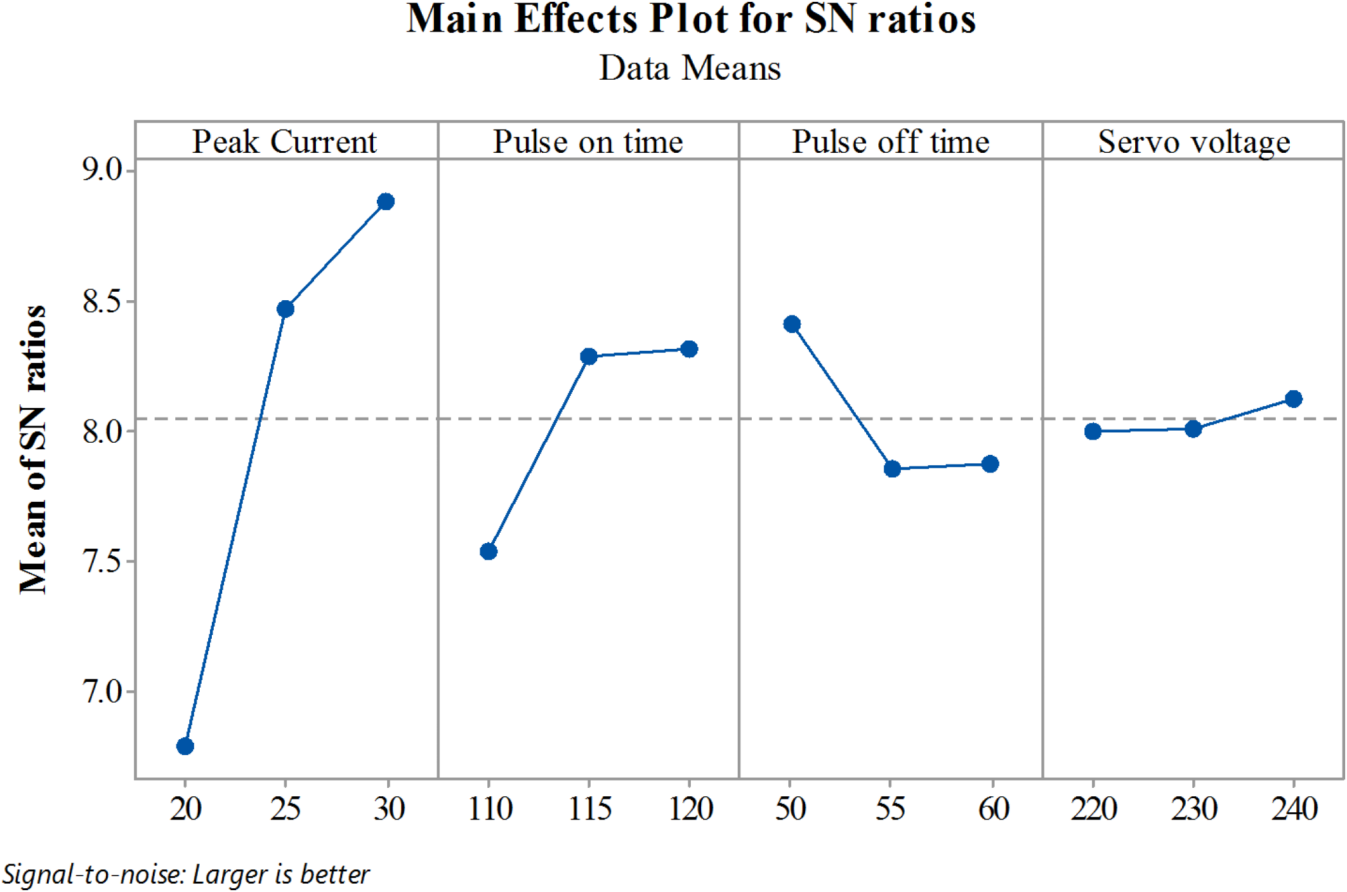
S/N Ratio graphs for the Surface Roughness.

The Surface Roughness (R_a_), corresponding signal-to-noise (S/N) ratio as well as ANOVA findings are shown in Tables 6 and 7, respectively. Surface roughness reduces with decreasing peak current (I_P_) as well as pulse on time (T_ON_), as seen by the S/N response graph in Fig. 5. The findings suggest that surface roughness in Ti-6Al-4 V Wire EDM is significantly influenced by peak current and pulse duration. The energy input during the machining process is influenced by these two parameters: peak current and pulse on time. The surface finish improves at lower energy input, which is attained by lowering peak current and pulse on time. This suggests that finer surface finishes can be achieved during the Ti-6Al-4 V WEDM process by modifying these parameters properly. The signal-to-noise (S/N) ratio is computed for each combination of factor levels. It is required to have a minimum, therefore smaller the better criteria are used. For smaller-the-better scenario, the S/N ratio is calculated using the following Eq. (3).

S/*N* = − 10 * log(Σ(Y^2^)/n) (3).

**Table 6:** Surface roughness and S/N ratio experimental results.

S. No	Peak Current	Pulse on time	Pulse off time	Servo Voltage	*R*_a_	S/*N*
1	20	110	50	220	2.14	−6.6082
2	20	115	55	230	2.19	−6.8088
3	20	120	60	240	2.23	−6.9660
4	25	110	55	240	2.47	−7.8539
5	25	115	60	220	2.66	−8.4865
6	25	120	50	230	2.84	−9.0663
7	30	110	60	230	2.56	−8.1647
8	30	115	50	240	3.01	−9.5713
9	30	120	55	220	2.79	−8.9120

**Table 7:** Experimental results of ANOVA for Surface Roughness.

Factors	Level 1	Level 2	Level 3	DOF	Sum of squares	Variance	Contribution
I_P_	6.7943	8.4689	8.8826	2	2.4454	1.2227	80.39
T_ON_	7.5422	8.2888	8.3147	2	0.3849	0.1924	12.65
T_OFF_	8.4152	7.8582	7.8724	2	0.2016	0.1008	6.62
SV	8.0022	8.0132	8.1304	2	0.0100	0.0050	0.29
Total				8	3.0419		100

## Conclusion

This research analyzes WEDM process parameters for machining Ti-6Al-4 V alloy using a Taguchi L9 orthogonal array design and analysis of variance. The peak current (Ip), pulse on time (TON), pulse off time (TOFF), and servo voltage (SV) are the input parameters that were addressed, with each being at three individual levels. The output responses measured are material removal rate (MRR) and the surface roughness (Ra). The experiments were conducted on EHT 10–30 A-2D Cutter CNC WEDM machine using a brass wire electrode and de-ionized water as a dielectric liquid. Results suggest that such variables as peak current and pulse on time are the two most critical factors that determine the level of MRR and Ra respectively, having 72.75% and 11.68% contributions to the results variations respectively. Using a peak current of 220 A, pulse on time of 11 µs, pulse off time of 50 µs and servo voltage of 20 V, it was discovered that the best surface roughness of 2.14 μm was possible on the wire electrode. The study advances the argument that adjusting the peak current and the pulse on time should be of primary concern in practice in WEDM of Ti-6Al-4 V alloy.

## Future research directions

Future research could explore additional wire EDM parameters such as wire tension, flushing pressure, and dielectric fluid type to optimize the machining of Ti-6Al-4 V alloy. Employing multi-objective optimization techniques like Genetic Algorithms (GA) or Particle Swarm Optimization (PSO) may enhance metrics such as MRR, surface roughness, tool wear rate, and energy consumption. Microstructural analysis might offer deeper insights into how EDM parameters influence material integrity, while predictive models using ANN or FEA could improve process control. Extending this research to materials like Inconel or tungsten carbide could generalize the findings. Assessing the environmental and economic impacts of different EDM parameters may also lead to more sustainable and cost-effective practices, contributing to the advancement of wire EDM technology.

## Data Availability

All data generated or analysed during this study are included in this published article.
